# Rapid analysis of metagenomic data using signature-based clustering

**DOI:** 10.1186/s12859-018-2540-4

**Published:** 2018-12-21

**Authors:** Timothy Chappell, Shlomo Geva, James M. Hogan, Flavia Huygens, Irani U. Rathnayake, Stephen Rudd, Wayne Kelly, Dimitri Perrin

**Affiliations:** 1 School of Electrical Engineering and Computer Science, Queensland University of Technology, 2 George Street, Brisbane, QLD 4001 Australia; 2 Institute of Health and Biomedical Innovation, Queensland University of Technology, 60 Musk Avenue, Kelvin Grove, QLD 4059 Australia; 3 School of Biomedical Sciences, Faculty of Health, Queensland University of Technology, 2 George Street, Brisbane, QLD 4001 Australia; 4 Queensland Facility for Advanced Bioinformatics (QFAB), Level 6 QBP (Bld 80), Chancellor’s place, The University of Queensland, St Lucia, Brisbane, QLD 4072 Australia

**Keywords:** Metagenomics, Clustering, Community analysis, Read signatures, Wound healing

## Abstract

**Background:**

Sequencing highly-variable 16S regions is a common and often effective approach to the study of microbial communities, and next-generation sequencing (NGS) technologies provide abundant quantities of data for analysis. However, the speed of existing analysis pipelines may limit our ability to work with these quantities of data. Furthermore, the limited coverage of existing 16S databases may hamper our ability to characterise these communities, particularly in the context of complex or poorly studied environments.

**Results:**

In this article we present the *SigClust* algorithm, a novel clustering method involving the transformation of sequence reads into binary signatures. When compared to other published methods, SigClust yields superior cluster coherence and separation of metagenomic read data, while operating within substantially reduced timeframes. We demonstrate its utility on published Illumina datasets and on a large collection of labelled wound reads sourced from patients in a wound clinic. The temporal analysis is based on tracking the dominant clusters of wound samples over time. The analysis can identify markers of both healing and non-healing wounds in response to treatment. Prominent clusters are found, corresponding to bacterial species known to be associated with unfavourable healing outcomes, including a number of strains of *Staphylococcus aureus*.

**Conclusions:**

*SigClust* identifies clusters rapidly and supports an improved understanding of the wound microbiome without reliance on a reference database. The results indicate a promising use for a *SigClust*-based pipeline in wound analysis and prediction, and a possible novel method for wound management and treatment.

## Background

Chronic wounds pose a significant risk to the patient, especially if the patient is elderly. Treatment and ongoing care are labour-intensive and costly, draining billions of dollars from public health budgets across the world. The impact of chronic wounds is expected to increase markedly as the population ages and as the incidence of type II diabetes increases in line with increased incidence of obesity. It is now well-established that bacterial populations in the wound may heavily influence the healing process [1]. The standard approach to partial characterisation of the wound microbiome is based on targeted sequencing of wound samples, followed by a lookup in a reference collection. This approach is hampered by the limitations of existing algorithms, by incomplete bacterial reference collections – with only a small fraction of species captured in curated and annotated databases – and by variability in the composition of bacterial communities. The increasing prevalence of sequencing in a clinical context will only exacerbate these concerns. Taxonomic classification of bacterial samples through Next-Generation Sequencing (NGS) remains challenging, with a relatively recent study noting that only a small fraction (<5*%*) of reads could be identified at the species level [2]. This is problematic for the clinician, as pathogenicity is usually species-specific – for example, a given *Escherichia* or *Bacillus* species may be a very dangerous pathogen, or completely harmless. New methods are needed to characterise microbiota without relying on existing sequence collections.

Massive reductions in sequencing costs mean that challenges now lie in the downstream computational analysis of genomic data at scale [3]. Routine collection and sequencing of wound samples will generate abundant data, and may lead to breakthroughs in our understanding of their biology and treatment. However, it will not be possible to deliver on this promise without methods capable of handling these large-scale datasets and rapidly identifying markers of healing or stagnation. Ideally, such algorithms will also be able to predict the progression of wound conditions over time.

This paper details a new method that relies on encoding the sequence reads as binary signatures to make clustering feasible at scale. Binary signatures are obtained from the k-mers contained within the reads. We show how the method can be used to characterise wound samples, and also demonstrate its general utility on previously published datasets. We evaluate the clusters obtained via the coherence of the entities in each cluster and the separation between individual clusters, and show that our approach generates clusters superior to a range of alternative approaches. We also consider the speed at which these clusters are produced.

To demonstrate the utility of our method, we analyse labelled patient data sampled over several weeks and show that tracking dominant clusters identifies markers of wounds refractory to treatment and markers of wounds that successfully heal. In this specific dataset, it is possible to identify with confidence those species likely to be responsible for the observed effect, but the method is more widely applicable. The cluster itself may be used as a proxy for bacterial species identification: proximity to a well defined cluster may support expectations of a similar clinical outcome. In such occurrences, the measured coherence of the cluster further reinforces our confidence in the prediction. It is important to note that such an approach differs significantly from the more common methods using database look-up for identification.

## Methods

### Sample preparation and Sequencing

Over a period of 12 weeks in 2011, 364 wound samples were collected (using the Z-technique [4]) from 56 patients undergoing treatment for a total of 66 chronic wounds at the Queensland University of Technology (QUT) wound clinic. These samples were collected by specialist wound care clinicians following a defined protocol for collection of Z-swabs and preserved at -80 ^∘^ C until DNA extraction was undertaken.

These wounds included mixed ulcers, arterial ulcers, venous ulcers, pressure ulcers, as well as amputation surgery, and were located at different areas of the lower extremities. All patients received standard wound care at the clinic, including silver, hydro-fibre, hydrogel and zinc paste dressings. Prior to swab collection, wounds were washed with water. It is acknowledged that the use of antimicrobial dressings is likely to have influenced the microbial flora. Wounds that did not heal after a period of *24 weeks* from initial presentation at the clinic were considered non-healing.

DNA was extracted from swab samples according to the protocol described by Price et al. [5], followed by physical and enzymatic lysis. Polymerase chain reaction (PCR) methods were then used to amplify the segments, using fusion primers derived from the universal 16S rRNA (prokaryotic small subunit ribosomal RNA). The samples were then sequenced using the Ion Torrent PGM platform [6], obtaining a total of 57,864,417 reads with an average length *l*=337. These reads were filtered by removing the barcode and primer, and reads with *l*<32 were discarded, reducing the read count to 46,313,157. Duplicate reads were also removed, further reducing the count to 24,892,382.

### Signature-based clustering

Use of *SigClust* allows us to identify tight groupings of structurally similar reads, which we can then use as a proxy for the original reads in the analysis of large datasets. We demonstrate the efficacy of our approach by verifying the method on previously published datasets of Illumina reads. Having shown that the clusters found are plausible, we then apply the method to the wound sample reads discussed above. We conjecture that the clusters so obtained will be linked to clinical outcomes for the patients who provided samples during the study. This potentially allows similarity-based inference to be conducted as new samples are obtained, without reference to an external database. In this section we describe the *SigClust* method and the experiments we used to verify its effectiveness.

*SigClust* makes use of a well-known clustering algorithm called *k-means* [7]. The approach involves starting with a random set of initial centroids, then progressively refining the centroids by moving them to the mean of the clusters they define, each time redistributing the points into the clusters to which they are closest. The approach is guaranteed to converge, usually to a local optimum, within a finite number of iterations; however, as the greatest gain to cluster coherency occurs in the earliest iterations [8], it is not usually necessary to iterate the method until it converges.

To calculate *k-means*, the underlying dataset must be represented as a set of vectors within a metric space; the distribution of points into clusters requires that the triangle inequality hold true, while the calculation of the centroids requires that an arithmetic mean be well-defined. An appropriate binary signature embedding is obtained through random indexing [9]. The resulting signatures support the properties required if we are to use *k-means*: distances between points can be calculated using a high speed bitwise (Hamming) distance calculation, and the mean of the cluster members can be computed with standard vector arithmetic. In addition, converting sequences into binary signatures provides a significant performance advantage for computing pairwise similarity scores: expensive alignment-based methods can be replaced by relatively inexpensive Hamming distance calculations, markedly reducing execution time for clustering methods that require a large number of comparisons.

The binary signatures created through random indexing function as a vector space representation of the underlying reads. These representations are fixed-length binary strings irrespective of the size of the reads, with the result that the number of cycles required to compare two signatures is identical for all signatures. This comparison is also very fast, as modern processors have dedicated instructions for both *exclusive or* and *population count*, which can be combined to compute efficiently the Hamming distance between these sequences.

The approach we use to generate signatures from text data is described in detail by Geva [10]. The main adjustment to this approach we have made here is that, rather than dividing the input document up into terms on whitespace boundaries as is common in text processing, we instead slide a k-mer window of length 5 over the input sequence. Upon reading each k-mer, we hash it to create a vector of pseudo-random values in the range [−1,+1]. This results in a total of *l*−4 vectors generated for each sequence of length *l*. These vectors are summed together and the resulting vector is quantised to create a binary signature. The quantisation process involves mapping negative values to 0-bits and non-negative values to 1-bits. Hence, a vector of length *w* will map to a signature consisting of *w* bits. The signature size is a configurable parameter for this approach: long signatures have the advantage of greater representational capacity, while shorter sequences trade this capacity for greater storage and processing efficiency. Informal experiments have shown that for datasets similar to the wound read set, a signature size of 256 bits offers a compact representation with only a modest decline in representation quality. Modern 64-bit processors support *exclusive or* and *population count* instructions, so the Hamming distance between two 256-bit signatures can be computed using only eight machine instructions.

One feature of the standard *k-means* algorithm is that the number of clusters *k* must be known a priori; the algorithm involves starting with an initial set of randomly chosen cluster centroids, then iteratively refining them. There is no provision for the number of clusters to change during this process. As a result, there is the possibility that a poor selection of *k* may result in a cluster arrangement that does not reflect the underlying dataset. In the absence of pre-existing knowledge of the structure of the dataset, the standard approach is to choose *k* through experimentation or heuristics. For the purposes of this study, we precisely choose the number of clusters we will receive as output in order to facilitate fair comparison with existing methods. Many of these alternatives do not allow the number of clusters to be specified a priori, but instead determine the number of clusters from a cluster similarity threshold supplied by the user. When comparing against multiple methods, we supply a value of *k* that ensures a fair comparison to other methods.

We summarise the full *SigClust* algorithm in Algorithm 1.


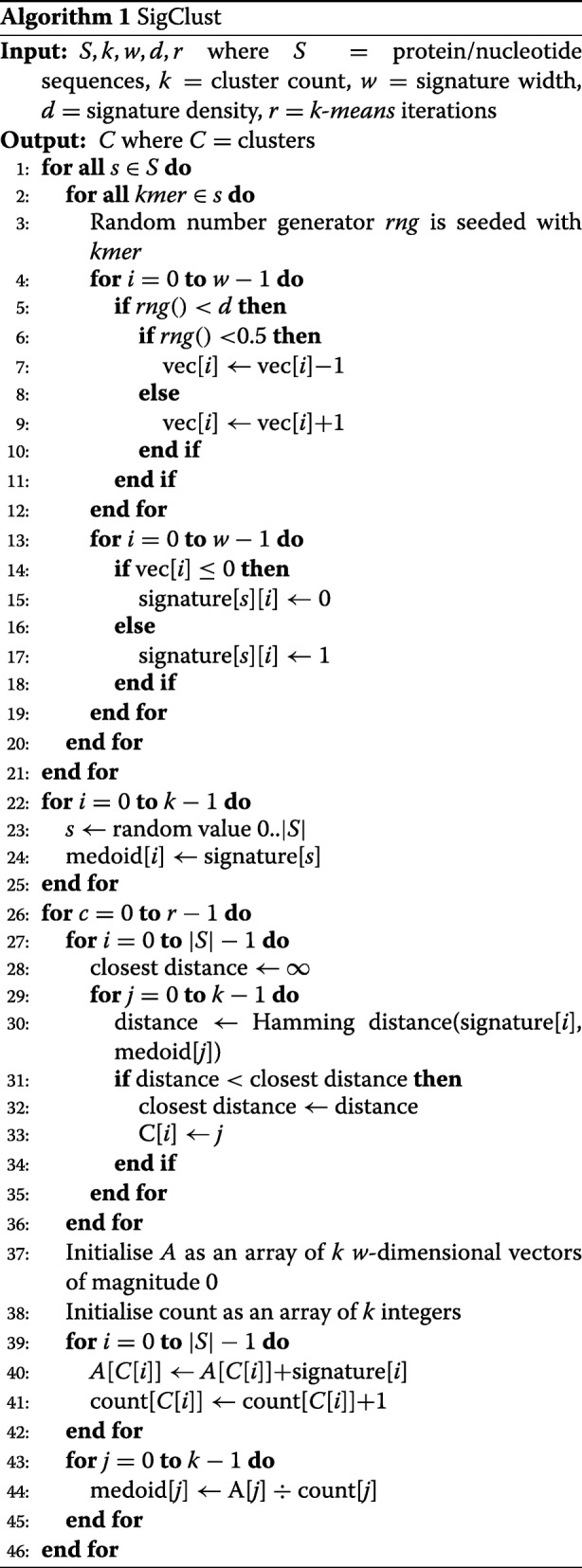


### Previously published datasets

*SigClust* was initially tested with two sizeable published datasets in order to demonstrate the general utility of the approach, and to allow for a direct comparison with methods proposed by other authors. The first collection, the *Oral Metagenome – Human MGP41* dataset, was introduced in [11]. This dataset consists of some 1237319 primer-targeted reads of the V5 region obtained by Illumina sequencing, with an average length of 59. A second, much larger collection, the *PRJEB4688 Evaluation Set*, was assembled for the analyses reported in [12]. Here we confine ourselves to the Illumina data, comprising 5497442 reads with an average sequence length of 253. Note that we examine these data sets purely from a computational perspective, and do not consider the biological significance of the clusters obtained, which lies outside the scope of this paper.

### Other clustering approaches

Our approach was compared to two alternative methods to benchmark its computational performance and the quality of the clusters obtained. *UClust* [13] employs a greedy heuristic based on similarity scores obtained via *USearch*. For each new sequence, *USearch* finds the closest existing centroid. If the identity score between this sequence and the centroid exceeds a certain threshold, the sequence is added to the cluster. Otherwise a new cluster is formed with this sequence as the centroid. *USearch* is a well known heuristic search method which rapidly identifies high-identity matches by counting the number of unique k-mers shared by the two sequences. Sequences with the highest counts are then examined in more detail, with identities now calculated using global alignments. This search process is terminated when a good enough match is found or when several rejections have occurred.

Our second baseline method relied on *BLAST* [14] to cluster the dataset against the NCBI complete 16S rRNA reference set [15]. Clusters were constructed by using *BLAST* to search for each wound read sequence in the 16S database using default *BLAST* settings, subsequently placing each sequence into a cluster associated with the matching 16S microbial strain, species or genus. This resulted in three different cluster sets at different levels of granuality; 9354 strain clusters, 8375 species clusters and 2221 genus clusters. Approximately 1 million (∼2*%*) of these searches could not be matched against any sequences in 16S and were excluded from the analysis. For the majority of searches the *E value* reported by *BLAST* was conclusive enough to show that the read did in fact belong to the corresponding strain; however, in other cases, only species or genus-level classification could be achieved.

As discussed in more detail below, we performed all computational experiments on a Linux workstation with 36 hyperthreaded Xeon cores running at 2.3GHz with 512GB RAM.

### Community analysis

Clusters obtained from the wound read collection were subsequently viewed as representative of the underlying microbial communities for each sample. As there may be changes in coverage when comparing across samples, we focused on the relative contribution of each of these clusters within the sample.

To analyse each wound, we tracked how these relative contributions changed dynamically over the sequencing period, and aimed to identify patterns in community structure that could be aligned with healing outcomes. This was achieved by focusing on the changes in community similarity over time. This *time-decay* method [16] was adapted from similar work on the decay of community similarity through spatial variation, or *distance-decay* [17, 18].

A quantitative measure of these variations in bacterial community structures was calculated with the *Bray-Curtis* (BC) dissimilarity score [19], using the *vegan* R package [20]. In our context, the BC dissimilarity is based on the cluster relative counts between different observations. For two distinct observation time points *i* and *j*, and clusters *x*_*k*_, it is defined as:

$${BC}_{ij} = \frac{{\sum\nolimits}_{k} \left|x_{ki}-x_{kj}\right|} {{\sum\nolimits}_{k} \left(x_{ki} + x_{kj}\right)} \in [0,1] $$

where each of the sums are indexed over the clusters. Note that the BC dissimilarity is not a proper metric: it does not satisfy the triangle inequality, and transitive relationships do not hold. This was not needed in our context: we just used evidence from the time-decay analysis and the relative cluster abundance to identify specific clusters and patterns of interest.

**Fig. 1:**
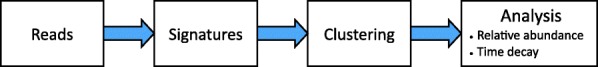
Clustering and analysis pipeline

The overall process from sample to community analysis is summarised in Fig. 1.

## Results

### Clustering results

To ensure a fair comparison between different clustering approaches, a metric that does not disproportionately favour one approach over another was required. In particular, metrics based on the Hamming distance between signatures may disproportionately favour *SigClust*, which works directly with these signatures, over methods which instead work directly with reads. Hence we make use of cluster quality metrics based on alignment scores between the original reads, adopting a sampling approach so that these metrics can be computed efficiently.

The process used for this comparative evaluation is based on global pairwise alignment with the *Needleman-Wunsch* [21] and local pairwise alignment with the *Smith-Waterman* [22] algorithms. Each method computes an alignment score for a pair of reads, though their resolution differs. To determine the overall level of cluster purity for a given arrangement, we sampled a large number of read pairs, pairs that share the same cluster (*intracluster pairs*) and pairs from different clusters (*intercluster pairs*). We were then able to compare the distributions of distances of the different categories of pairs, allowing us to compare the respective cluster purities for each clustering approach.

**Fig. 2:**
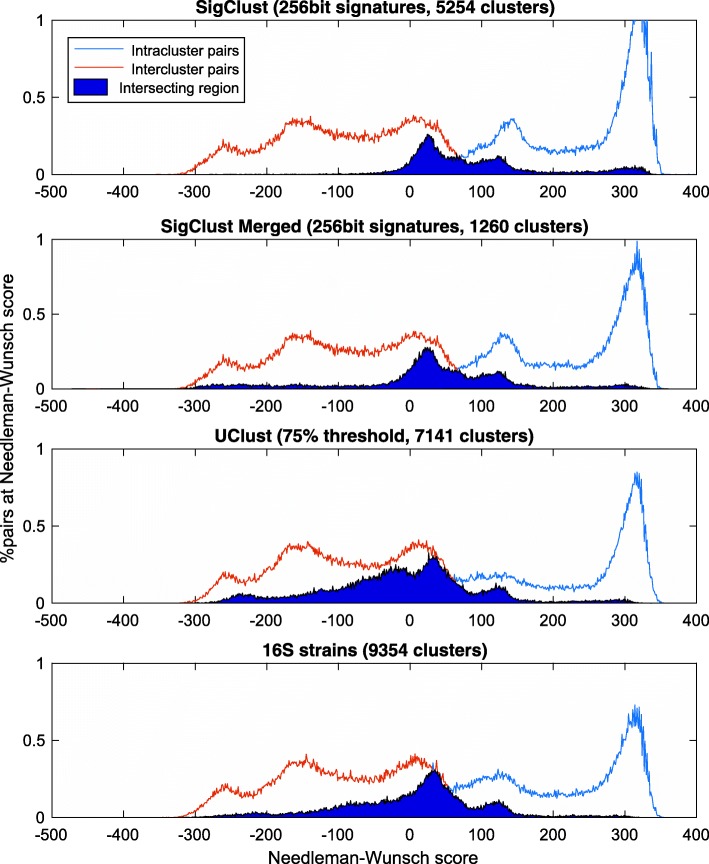
Needleman-Wunch global alignment cluster analysis. Histogram of *Needleman-Wunch* scores between random pairs of reads in the same cluster (intracluster pairs) and pairs of reads from different clusters (intercluster pairs)

Comparisons between *SigClust*, *UClust* and the *BLAST*-16S based method are considered below. Distributions of pairwise *Needleman-Wunsch* scores are depicted in Fig. 2, showing a clear advantage for *SigClust*. Separation between the distributions is more pronounced for *SigClust* than for the baseline methods, notwithstanding the smaller number of clusters generated by our approach; due to the nature of the calculation, arrangements involving a larger number of clusters have an advantage when it comes to cluster purity. Table 1 shows the mean alignment scores for each approach, along with the execution time required, demonstrating that *SigClust* offers substantial performance advantages over the other methods.

**Table 1:** Comparison of clustering methods based on *Needleman-Wunsch* alignment scores

Wound Microbiome						
46313157 sequences, average sequence length: 337						
Method	Time	Clusters	Intercluster		Intracluster	
	(m)		Avg	SD	Avg	SD
SigClust	16	5254	-68.8	125.1	219.4	107.7
Merged SigClust	18	1260	-73.9	118.0	180.9	136.9
UClust T=0.75	156	7141	-81.4	111.6	148.5	142.1
16S Genus	2586	2221	-82.4	104.6	98.6	167.4
16S Species	2586	8375	-76.9	110.8	125.6	162.5
16S Strains	2586	9354	-82.4	104.6	98.6	167.4
Oral Metagenome – Human (mgp41)[11]						
1237319 sequences, average sequence length: 59						
Method	Time	Clusters	Intercluster		Intracluster	
	(m)		Avg	SD	Avg	SD
SigClust	0.2	17621	5.5	22.3	51.6	14.1
UClust T=0.75	1.7	17621	-4.4	14.4	38.8	13.3
PRJEB4688 [12]						
5497442 sequences, average sequence length: 253						
Method	Time	Clusters	Intercluster		Intracluster	
	(m)		Avg	SD	Avg	SD
SigClust	1.62	6998	-94.8	126.6	250.1	77.2
UClust T=0.75	9	6998	-109.0	117.4	121.5	93.5

Results are shown for the wound data, and for two previously published Illumina metagenomic datasets. We report for each method the clustering time in minutes and the number of clusters returned. The remaining columns of the table show the mean and standard deviation of the separation for the sampled intercluster and intracluster pairs

**Fig. 3:**
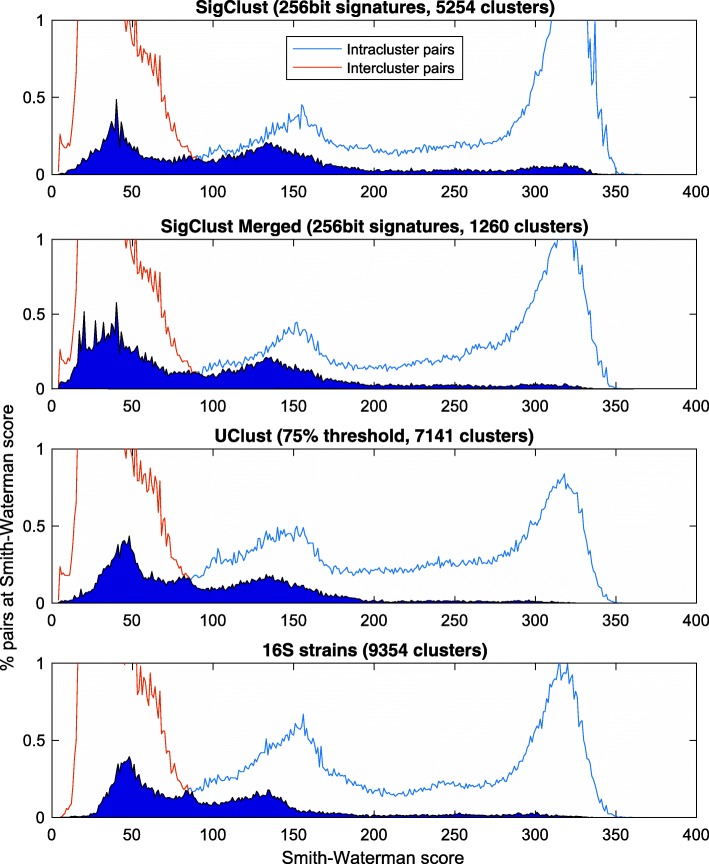
Smith-Waterman global alignment cluster analysis. Histogram of *Smith-Waterman* scores between random pairs of reads in the same cluster (intracluster pairs) and pairs of reads from different clusters (intercluster pairs)

The same evaluation was repeated with *Smith-Waterman* scores with the outcomes shown in Fig. 3, where the advantages are less clear. As is shown in Table 2, *SigClust* provides the greatest distance between the average intracluster and intercluster scores, yet the degree of overlap in the histograms remains large, suggesting the clusters are not as well separated.

**Table 2:** Comparison of clustering methods based on *Smith-Waterman* alignment scores

Wound Microbiome						
46313157 sequences, average sequence length: 337						
Method	Time	Clusters	Intercluster		Intracluster	
	(m)		Avg	SD	Avg	SD
SigClust	16	5254	59.0	58.7	234.4	97.9
Merged SigClust	18	1260	55.5	51.2	211.6	104.9
UClust T=0.75	156	7141	50.3	41.8	202.9	95.0
16S Genus	2586	2221	43.9	28.0	188.9	92.5
16S Species	2586	8375	48.7	39.6	205.8	93.6
16S Strains	2586	9354	50.1	42.6	206.7	93.5
Oral Metagenome – Human (mgp41)[11]						
1237319 sequences, average sequence length: 59						
Method	Time	Clusters	Intercluster		Intracluster	
	(m)		Avg	SD	Avg	SD
SigClust	0.2	17621	25.5	11.8	53.7	9.6
UClust T=0.75	1.7	17621	20.2	6.9	43.4	9.1
PRJEB4688 [12]						
5497442 sequences, average sequence length: 253						
Method	Time	Clusters	Intercluster		Intracluster	
	(m)		Avg	SD	Avg	SD
SigClust	1.62	6998	44.4	48.5	257.7	67.0
UClust T=0.75	9	6998	37.1	38.0	159.1	66.9

As before, results are shown for the wound data, and for two previously published Illumina metagenomic datasets. We report for each method the clustering time in minutes and the number of clusters returned. The remaining columns of the table show the mean and standard deviation of the separation for the sampled intercluster and intracluster pairs

The discrepancy here can partially be explained by observing that the current approach of generating a single binary signature for each read is ultimately global, and unable to reward perfect matching of highly similar subsets in the same way as local alignment methods such as *BLAST*. As a result, while we can present the *SigClust* algorithm as a highly capable global clustering tool, if local clustering is more desirable for a particular application and local sensitivity is of the utmost importance, there may be more suitable tools available.

The same experiment was repeated using two published datasets of reads sequenced with Illumina technology: the *Oral Metagenome - Human (mgp41)* [11] study and *PRJEB4688* [12]. The *Needleman-Wunsch* results for these datasets are included in Table 1, while the *Smith-Waterman* results are included in Table 2. To ensure a fair comparison, we compared *SigClust* against *UClust* directly, using the same number of clusters reported by *UClust* as input into *SigClust*. The results show *SigClust* continues to be competitive with *UClust* while taking a fraction of the time to run. The difference in cluster purity between the two methods is more marginal with the *Oral Metagenome* dataset, potentially due to the shorter reads reducing the advantages of *SigClust*’s fixed-length encoding. For the larger *PRJEB4688* dataset, *SigClust*’s advantages are further emphasised, with the approach offering very significant performance improvements along with a clear gap in the distributions between intracluster and intercluster pairs. This shows that at least some of *SigClust*’s advantages are portable to widely varying datasets across different sequencing technologies.

### Biological significance

In this section we undertake community analysis of the clusters obtained via *SigClust*, using the approach described above. As the value of *k* selected for algorithm evaluation (obtained empirically from *UClust*) is relatively large, proximal clusters were merged. This process is equivalent to choosing a lower value of *k* when executing *SigClust*, and reduces the reliance on the exact value for *k*.

The choice of merge proximity threshold is data-specific, and a natural intra-cluster distance may be inferred through experiment. For the wound reads, a Hamming distance of 35 provides a suitable threshold for cluster merging, corresponding to a similarity score of approximately 95*%*−97%, although this varies somewhat by read (or corresponding species or strain). This threshold distance choice for clusters is grounded in the properties of the wound reads and their respective species and strains.

Armed with a threshold value, cluster merging proceeds as follows: We create a binary matrix *M* recording cluster pairs identified as potential merge candidates based on the Hamming distance between them. Here *M*[ *i*][ *j*]=1 if clusters *i* and *j* are candidates for a merge; otherwise *M*[ *i*][ *j*]=0. Clusters are sorted in decreasing order of their number of potential partners. For each cluster, we extract potential partners to form a submatrix *M*^′^, which is then processed to select merges which favour cluster coherence. We go through all potential partners and reject the one that has the fewest shared partners (i.e. the row with the lowest sum). We repeat this process until all rows only contain 1s (in which case the remaining clusters are merged together) or alternatively, all partners have been rejected and no merge operation is performed. If a merged cluster is created, the individual member clusters are not considered as potential partners for the following clusters.

**Fig. 4:**
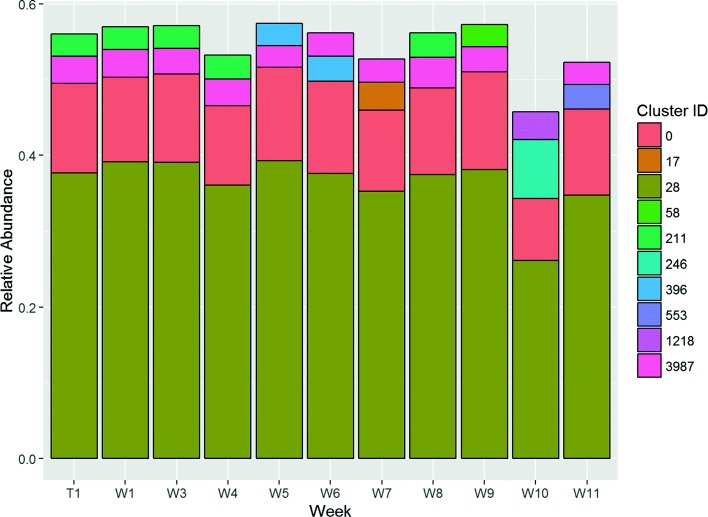
Relative Cluster Abundance for wound #4059

**Fig. 5:**
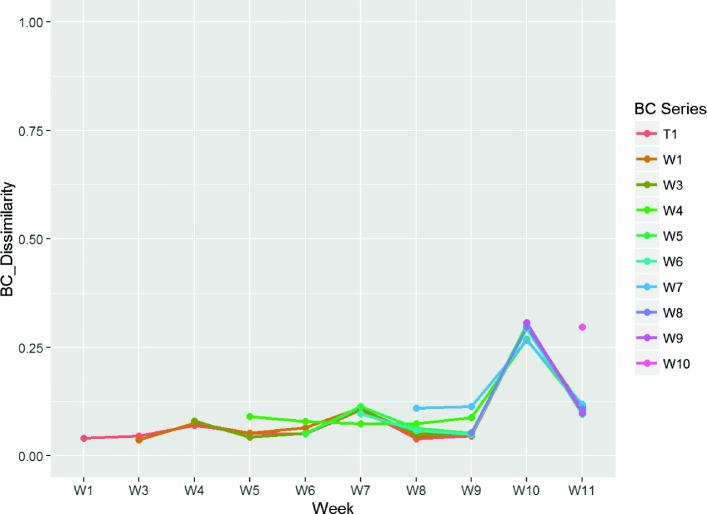
Bray Curtis dissimilarity analysis for wound #4059. Each series shows the variation in BC dissimilarity for each time point *relative* to the observation immediately before, commencing with the time point following the label. So, label *W4* shows observations for *W5* – relative to *W4*, for *W6* – relative to *W5*, and so on. For *W11*, we see only the single observation at *W12*, relative to *W11*

The clusters obtained reveal patterns that are associated with wound healing outcomes. We have identified a set of clusters present at high levels in a sample time-series – multiple wound samples collected from patients over a 12 week period. Of all the clusters identified by *SigClust*, five are present in 20 of the 24 non-healing wounds. These clusters, and their dominant species, are listed as follows: 0,34,50 (*Staphylococcus aureus*), 28 (*Enterococcus faecalis*), and 80 (*Bacteroides fragilis*). Wound *#*4059, which is a typical non-healing representative (Fig. 4), shows dominance of clusters 28 (*E. faecalis*) and 0 (*S. aureus*) over the entire sampling period. Their persistence in the wound is apparent when looking at the BC dissimilarity (Fig. 5).

Abundant species of this nature may be identified readily using our methods or through a variety of well-established alternatives such as *Mothur*/*QIIME*. In earlier sections of this paper we observed that *SigClust* rapidly yields clusters notable for their strong internal coherence and clear inter-cluster separation. *SigClust* may thus offer significant advantages over existing clustering approaches when characterising diverse communities, especially those comprising taxons exhibiting low abundance. If clusters are more coherent and distinct, then we may have greater confidence in their utility as operational units, and in subsequent identifications made through sampling of their constituent members.

**Fig. 6:**
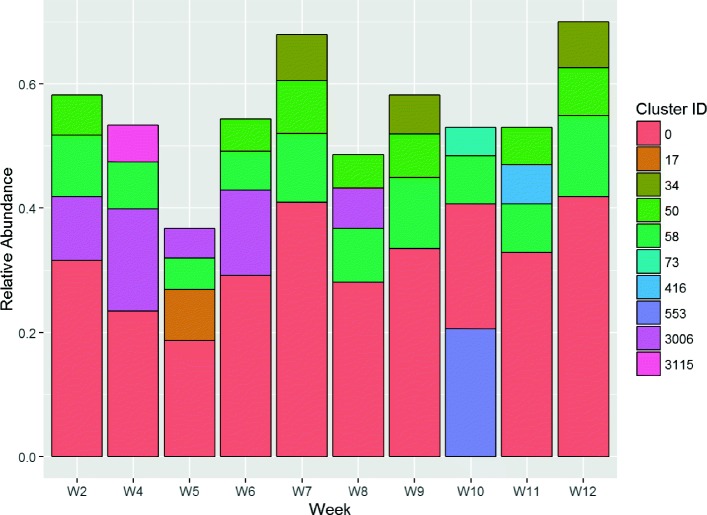
Relative Cluster Abundance for wound #4032

**Fig. 7:**
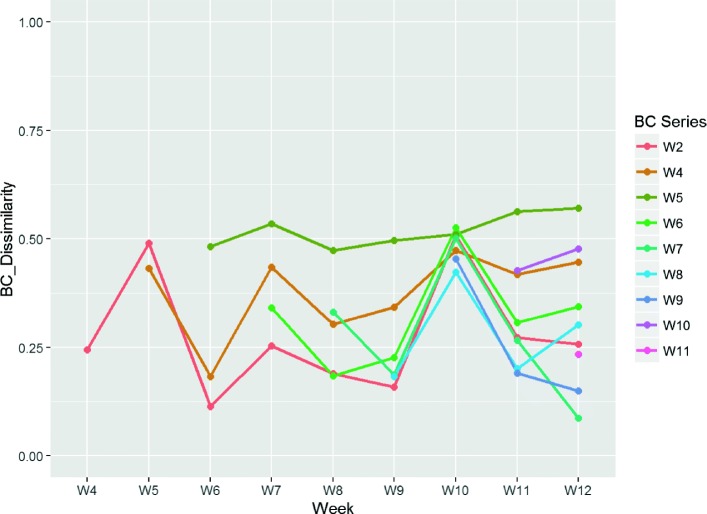
Bray Curtis dissimilarity analysis for wound #4032. Each series shows the variation in BC dissimilarity for each time point *relative* to the observation immediately before, commencing with the time point following the label. See the caption for Fig. 5 for a more detailed explanation

The five clusters listed above for non-healing wounds are also present in most of the wounds that took at least 12 weeks to heal (some 18 out of 20 such wounds). Wound #4032 (Fig. 6) is a good representative example of such wounds. Note that the time points are measured relative to the first clinic visit, rather than as an absolute time since the wound first occurred. As a result we only have a lower bound of the total healing time for these wounds. In the earlier study that produced this dataset, wound sampling was limited in all cases to a maximum of 12 weeks after the first visit. Subsequent time points are not available, but a possible interpretation of the eventual outcome is that the wounds healed once these clusters were no longer present. The BC dissimilarity results for wound #4032 (Fig. 7) support this hypothesis. Over the entire sampling period the community structures seem less stable. By week 12, only clusters characterised as *S. aureus*) remain (clusters 0,34,50 and 58). This is consistent with the interpretation that the wound was unable to heal at this stage and required future clearing of these clusters.

**Fig. 8:**
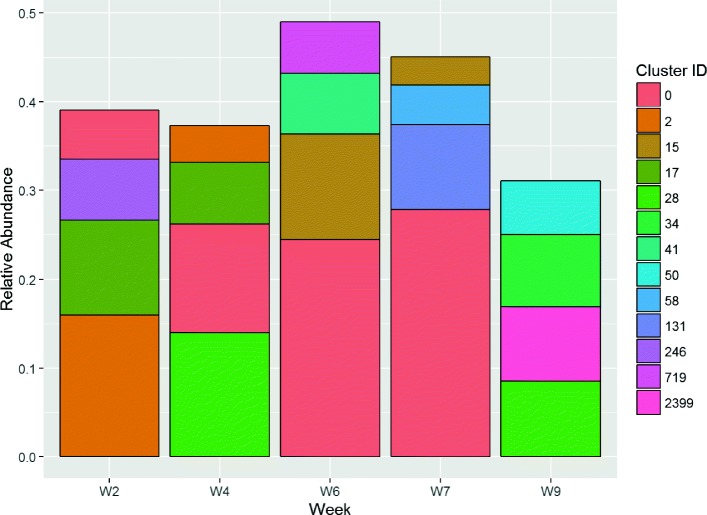
Relative Cluster Abundance for wound #4068

This interpretation is further supported by other wounds such as *#*4068 (Fig. 8). For this wound, clusters 0 and 28 have a significantly reduced contribution to the overall population by week 9, and the wound was observed to heal by week 12. These clusters are identified as *S. aureus* and *E. faecalis*, respectively.

**Fig. 9:**
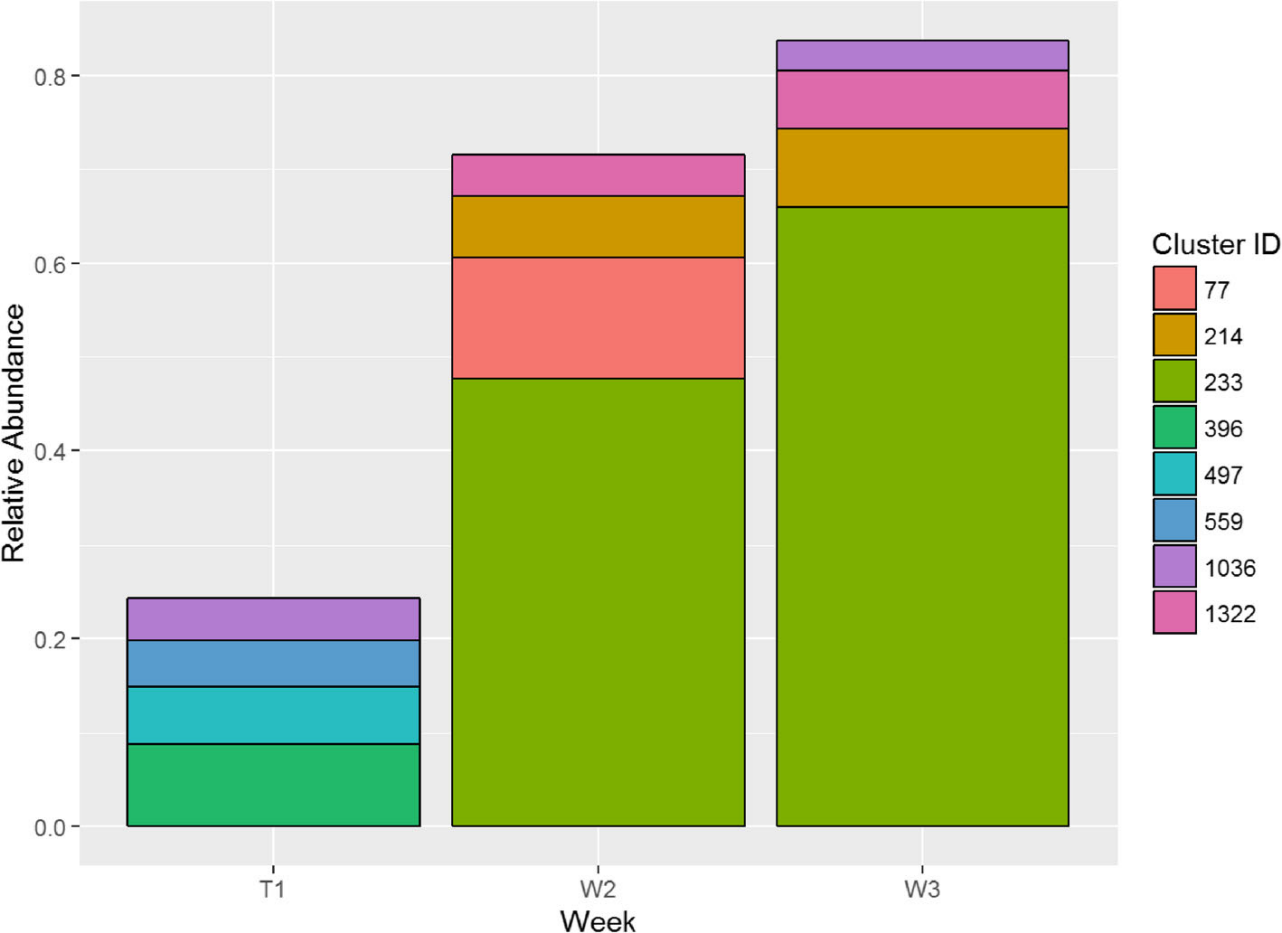
Relative Cluster Abundance for wound #4046

On the other hand, wounds that healed rapidly (requiring four weeks or less) generally contained different clusters that were not present at significant levels in non-healing wounds. For instance, cluster 233 is present in wound *#*4046 (Fig. 9), but did not appear elsewhere. One wound healed in just four weeks despite still showing a peak in cluster 0 (*S. aureus*) in week 3. However, that wound exhibits a very high BC dissimilarity, which suggests that the microbial population was unstable. This therefore remains consistent with our earlier interpretation.

Taken together, these results demonstrate that our method can track fluctuations in the wound microbiome over the sampling period and detect the presence of pathogenic bacteria in some of the wounds and samples. They also show that it is possible to link specific clusters with healing or non-healing outcomes for these wounds.

### Computational performance

Table 1 shows the execution times (in minutes) for *SigClust* and the baseline methods *UClust* and *BLAST* over the wound reads dataset, the *Oral Metagenome - Human (mgp41)* [11] Illumina dataset and the *PRJEB4688* [12] Illumina dataset. These experiments were run on an Intel Xeon EE5-2699 v3 with 36 hyper-threaded cores clocked at 2.30GHz, for a total of 72 hardware threads. Both the signature generation code and the clustering code were multi-threaded with OpenMP and configured to consume all available threads. The time of 16 min for the standard *SigClust* approach on the wound dataset includes both signature generation time and clustering time. Generating 256-bit signatures for each of the 24,892,382 reads that remained after removing short sequences and duplicates took 4 min and 28 s of elapsed wall time. Each iteration of *k-means* required an average of 2 min and 51 s, and we ran a total of four iterations, yielding a clustering time of 11 min and 24 s. The Merged *SigClust* approach used the same process, but also included an additional two minutes to generate the mutual distance graph between the clusters and to find close clusters to be merged.

*UClust* was run on the same hardware; however, it was not able to make full use of all the hardware threads and so did not exhibit a similar speed-up. The difference between *UClust* and *SigClust* comes close to a 10× performance increase, depending on the size of the dataset, with larger datasets showing the greatest difference. We see this on both the wound dataset, some 16 min vs. 156 min, as well as on the published Illumina datasets, with 0.2 min vs. 1.7 min on the *Oral Metagenome* study and 1.62 min vs. 9 min on *PRJEB4688*.

The *BLAST* runs were also executed on the same hardware, but multi-threading here provided only limited advantages due to the nature of the algorithm, with *BLAST* unable to take full advantage of the set of threads available. *BLAST* was not able to offer performance competitive with the other approaches.

## Discussion

Ongoing technological advances and consequent reductions in the cost of DNA sequencing may potentially revolutionise clinical microbiology, but sample processing and analysis is not yet straightforward, and may still require significant specialist bioinformatic expertise [23–25]. Millions of prokaryotic species exist in nature [26, 27] and less than 0.2*%* of them have been identified, significantly limiting our understanding of the role of bacteria in human health and disease. Despite enormous progress in the study of bacterial organisms over the past decade or more, there remains no comprehensive database listing all bacterial species associated with humans [28]. Such a database would provide crucial advantages in healthcare, allowing clinicians to link the pathological changes observed in their patients with potentially causative bacterial species.

Bacteria colonize all wounds whether they are chronic or acute. Currently, there is an increased interest in investigating whether there is a correlation between different bacterial communities in wounds and the ultimate repair of the wound, i.e. whether bacteria contribute to the maintenance of a wound in a chronic state. Chronic wounds are defined as lasting more than three months and occur most commonly in diabetic, elderly and immunocompromised people [29–31]. Given the economic burden and more importantly, the significant morbidity caused by slow healing wounds, a “personalised medicine" approach for examining and treating these chronic wounds could result in a better way forward for wound management and patient prognosis [31].

Yet there remain a number of computational and analytical challenges to be overcome before this vision is realised. For example, identification of bacterial determinants of wound healing and its absence can be compromised by selection bias in the experimental protocol. The 16S rRNA gene sequence analysis pipeline typically consists of three main components: pre-processing of sequences, constructing Operational Taxonomic Units (OTUs) that are similar to bacterial taxa or species, followed by annotation of the OTU tables. Pre-processing the 16S rRNA is used to remove low-quality sequences prior to the construction of the OTU table and chimeric sequences (generated during the PCR amplification process) are identified and removed from the dataset [32]. In this step, significant proportions of 16S rRNA sequences are eliminated and hence can lead to selection bias of bacterial species representation in the sample. Importantly, the appropriate analysis methods and parameters used for 16S rRNA sequence analysis are dependent on the method used for sequencing as well as the region of the 16S rRNA gene targeted for sequencing [33].

Clustering – the main focus of this paper – is an essential step in existing workflows. After pre-processing, the OTU table is constructed by clustering similar sequences based on a defined similarity threshold. Several approaches are commonly used for this purpose [34], and each may have a marked effect on the resulting analysis. The choice of reference clusters and the similarity thresholds employed are both known to affect the outcome significantly [35–37]. Issues resulting from variations in sequencing depth can be addressed through normalisation and rarefaction [38]. However, most approaches rely on a specific database collection, with annotation of the OTU table based on representative taxonomic and phylogenetic relatedness [39, 40]. Moreover, a number of alternative approaches are used to classify 16S rRNA gene sequences, including *BLAST*, *RDP* (a k-mer based method) and *phylogenetic placement* [41], where phylogenetic trees are generated and used for diversity metrics (eg. UniFrac) or for data visualization.

The underlying approach described in this paper aims to handle the common situation where a complete sequence reference list is unavailable. Instead, we rely on read clusters to act as the operational units. In some cases, clusters may be resolved to known references using traditional methods. While this is not a necessary condition for our approach, it does increase our confidence in its utility and practicality in identifying relevant clusters. We therefore looked to verify clusters of interest by determining the dominant bacterial strain in the NCBI 16S rRNA reference sequence set. We used the NCBI *BLAST* tool for this purpose. Each query consisted of 100 randomly-selected reads from a given cluster. The most commonly selected strain among this read sample was then associated with the cluster, together with the fraction of reads that returned that strain. This fraction was used to approximate the prevalence of the strain within the cluster.

Based on this approach, clusters 0,34 and 50 were associated with *Staphylococcus aureus* (subsp. anaerobius strain MVF-7). It is widely known that Staphylococcal biofilms may limit wound healing [42], and this is identifiably associated with the healing outcomes observed. *Enterococcus faecalis* (strain NBRC 100480; cluster 28) also impairs wound healing [43]. Equally interesting is the appearance of *Bacteroides fragilis* (strain NCTC 9343) in cluster 80. *Bacteroides* species are normally commensal gut organisms but they may also be responsible for certain types of infection, and *B. fragilis* is unique in inducing abscess formation as the sole infectious agent [44, 45], with injection of capsules proving sufficient [46]. Compromised wound healing is thus to be expected.

These findings are supported by an earlier study using standard methods [47], providing clear evidence that *SigClust* can produce biologically relevant clusters and contribute to our understanding and treatment of chronic wounds. Rapid assessment of microbial diversity allows for tailored antimicrobial therapy to be administered in a timely fashion. Targeted approaches of this nature limit overuse of broad-spectrum antimicrobials and reduce the likelihood that antimicrobial resistance may develop.

While we have successfully employed 16S reference sequences to validate our methods, we should emphasise that the utility of our approach does not rely on this reference. The prevalence of healthcare-related bacterial studies means that most wound-related bacteria may be found in the NCBI 16S database, but our method is more general and may be applied when database coverage is poor or non-existent. Analysis here is not dependent on the existence of a reference database, but requires only that some ground truth be available from the domain of interest, allowing us to associate information with each cluster, supporting its application across a wide range of scientific and clinical contexts. The utility of our methods is further enhanced by their inherent parallelism and the reduced memory footprint and extremely rapid pairwise comparisons that come with the signature-based representation. The use of binary signatures allows the approach to scale to very large collections beyond the scope of competing methods.

Tables 1 and 2 report the application of *SigClust* to previously published Illumina datasets. While we have not performed community analysis on the clusters obtained, these results showcase the general utility of the method, the quality of the clusters produced and the consistent performance advantages of the algorithm over *UClust*, a tool widely known for its computational efficiency.

## Conclusions

In this paper we have introduced *SigClust*, a novel, high-speed clustering approach which allows the accurate analysis of read collections at scale, potentially supporting the timely processing of clinical wound samples as part of an integrated pipeline. We have further demonstrated the utility of the approach through community analysis, highlighting the correlation of certain cluster types with wounds that heal successfully and of others with wounds refractory to treatment. These findings have been further validated through 16S reference lookup and their alignment with the outcomes of an earlier, independent study of the same dataset. We note further that the performance advantages that underpin our success continue to hold even when the method is applied to large-scale datasets with markedly different characteristics based on very different sequencing technologies.

The superior clustering performance offered by these methods, along with their computational efficiency, will allow more rapid progress in our understanding of wound microbiota and in the development of better diagnostic and therapeutic approaches for non-healing wounds. As the method may operate in the absence of an external reference database, there is wide potential for its application across a range of metagenomic domains, and its suitability for very large scale collections will make it a natural candidate for these analyses as the availability of metagenomic datasets continues to grow rapidly.
